# Barriers to colorectal cancer screening in community health centers: A qualitative study

**DOI:** 10.1186/1471-2296-9-15

**Published:** 2008-02-27

**Authors:** Karen E Lasser, John Z Ayanian, Robert H Fletcher, Mary-Jo DelVecchio Good

**Affiliations:** 1 Department of Medicine, Cambridge Health Alliance/Harvard Medical School, Cambridge, MA, USA; 2 Division of General Medicine, Brigham and Women's Hospital, Boston, MA, USA; 3 Department of Health Care Policy, Harvard Medical School, Boston, MA, USA; 4 Department of Ambulatory Care and Prevention/Harvard Medical School and Harvard Pilgrim Health Care, Boston, MA, USA; 5 Department of Social Medicine/Harvard Medical School, Boston, MA, USA

## Abstract

**Background:**

Colorectal cancer screening rates are low among disadvantaged patients; few studies have explored barriers to screening in community health centers. The purpose of this study was to describe barriers to/facilitators of colorectal cancer screening among diverse patients served by community health centers.

**Methods:**

We identified twenty-three outpatients who were eligible for colorectal cancer screening and their 10 primary care physicians. Using in-depth semi-structured interviews, we asked patients to describe factors influencing their screening decisions. For each unscreened patient, we asked his or her physician to describe barriers to screening. We conducted patient interviews in English (n = 8), Spanish (n = 2), Portuguese (n = 5), Portuguese Creole (n = 1), and Haitian Creole (n = 7). We audiotaped and transcribed the interviews, and then identified major themes in the interviews.

**Results:**

Four themes emerged: 1) Unscreened patients cited lack of trust in doctors as a barrier to screening whereas few physicians identified this barrier; 2) Unscreened patients identified lack of symptoms as the reason they had not been screened; 3) A doctor's recommendation, or lack thereof, significantly influenced patients' decisions to be screened; 4) Patients, but not their physicians, cited fatalistic views about cancer as a barrier. Conversely, physicians identified competing priorities, such as psychosocial stressors or comorbid medical illness, as barriers to screening. In this culturally diverse group of patients seen at community health centers, similar barriers to screening were reported by patients of different backgrounds, but physicians perceived other factors as more important.

**Conclusion:**

Further study of these barriers is warranted.

## Background

Colorectal cancer is the second leading cause of cancer death in the US. In 2007, an estimated 153,760 people will be diagnosed with colorectal cancer, and it is estimated that 52,180 will die of the disease [1]. Despite the availability of effective screening tests [2-6] a large proportion of Americans are still not being screened for colorectal cancer [7-9]. Patients at greatest risk of not being screened include racial and ethnic minorities [9,10], patients with Medicaid or no health insurance [7,11,12], the foreign born [11,13], and patients with low socioeconomic status [14] – groups that are commonly served by community health centers [7,15].

Researchers have identified and explored numerous barriers to colorectal cancer screening [7,16]. Quantitative studies have found the following to be barriers specific to poor and underserved populations: demographic factors (insurance, social class, and race/ethnicity) [12,17-21], language, embarrassment, lack of knowledge about colorectal cancer screening [22] culture-specific beliefs [10], and level of acculturation [23]. Qualitative studies have characterized such barriers in more detail, and have sought to answer the question of why poor and minority patients are not being screened. Such studies have included white, African-American [24], Latino and Chinese patients [25], and have found fear (of both pain and of discovering cancer), shame of being seen as sick or weak, feelings of violation, mistrust, and fatalism to be barriers to screening. Patients also reported that they did not know where to obtain screening, and had difficulty in obtaining an appointment for screening [25]. Yet prior qualitative studies have not included patients from Brazil, Portugal, the Azores, Cape Verde or Haiti, large immigrant groups in Massachusetts and elsewhere in the US, and none have simultaneously interviewed each patient's primary care provider.

Our study had two primary objectives: 1) to identify and describe barriers to and facilitators of screening in an ethnically diverse population of patients served by community health centers and 2) to compare patients' and physicians' views regarding the reasons why patients did not receive screening. We used qualitative methods to obtain in-depth information from patients and their physicians.

## Methods

### Setting and participants

Cambridge Health Alliance is a regional healthcare system with three hospitals and more than twenty primary-care centers in Cambridge, Somerville, and Everett, MA. Cambridge Health Alliance also includes the Cambridge Public Health Department and Network Health, a managed Medicaid plan. Designated by the Agency for Health Care Quality and Research (AHRQ) as a Primary Care Practice-Based Research Network, the health centers predominantly serve a multi-cultural, low-income population.

At the time of the study, January 2005–December 2006, most of the health centers used a limited electronic clinical data system (Meditech) that included patient demographics, medical visit information, and diagnostic test results. There was no organized screening program in place at the time of the study; patients were offered screening on an ad-hoc basis during primary care visits. Among patients receiving care at 8 different community health centers, we identified patients aged 52–80 who appeared to be unscreened for colorectal cancer. Since the database did not capture diagnostic tests performed outside of the health center network, we anticipated that some patients who appeared unscreened would in fact have received colorectal cancer screening. We included these patients in our study as we sought both to understand barriers to screening as well as factors that facilitated screening. We based eligibility for colorectal cancer screening on a modified version of the most recent HEDIS measure [26]. The denominator of the measure included any patient aged 52–80 who had one visit to a primary care physician in a community health center in each of the 2 previous years. The numerator of the measure included any patient who received colonoscopy in the past 10 years, sigmoidoscopy or barium enema in the past 5 years, or fecal occult blood testing (FOBT) during each measurement year.

We limited our sample to patients who spoke English, Portuguese, Portuguese Creole, Spanish and Haitian Creole, and whose primary care physicians had the most patients in the age range of interest (52–80). This sample included 301 patients. We then e-mailed the 13 primary care physicians of these patients and invited them to participate in the study; all agreed to participate. All patients in the study were cared for by one or more of the primary care physicians in the study. We thus interviewed a convenience sample of patients and primary care physicians, excluding patients with active substance abuse (where, according to the physician, the patient would not be able to participate in the interview in a meaningful way), a history of colorectal cancer, or mental retardation. The Institutional Review Board at the Cambridge Health Alliance approved the study protocol. All participating patients and physicians provided written informed consent.

### Data collection

We conducted semi-structured individual interviews with patients to obtain in-depth information about why patients who were eligible for colorectal cancer screening had or had not been screened, respectively. For unscreened patients, we also interviewed each patient's physician to gain their perspective regarding the reason their patient had not been screened. Since our goal was to obtain a broad range of information to understand patients' screening decisions, we included both screened and unscreened patients. Known as "maximum variation sampling," this sampling approach is used in qualitative research to encompass a broader variety of perspectives [27].

A primary care physician (KEL) and a medical sociologist with expertise in qualitative research (MG) developed an open-ended, semi-structured interview instrument to explore subjects' experiences with colorectal cancer screening. The investigators piloted the interview instrument with patients, and revised the instrument accordingly. In developing the instrument, we reviewed the extensive ethnographic colorectal cancer screening literature to ensure that our instrument encompassed barriers and facilitators encountered by other researchers working with comparable underserved and deprived patient populations [24,28]. Our interview instrument is available as Additional file 1.

We delivered probes in an order that was based on how the interview unfolded. The initial portion of the instrument elicited pertinent aspects of the subject's cultural, family, and educational history. We also assessed specific cognitive knowledge of colorectal cancer and the role of colorectal cancer screening. In the second portion of the interview, we asked participants to recall and to describe in detail their experience discussing colorectal cancer screening with the person they identified as their primary care physician. We probed participants about logistical barriers to obtaining screening (lack of transportation, inability to take time off from work, child care responsibilities), health beliefs (that colorectal cancer screening is harmful, unhelpful, painful), psychological symptoms or conditions that may relate to completing screening (fears of cancer, fear of leaving one's home or riding on public transportation, inability to keep appointments due to depression, PTSD symptoms related to prior sexual abuse), distrust of the medical system (paranoia, feeling of being used as a "guinea pig"), fatalism, social stressors (financial and housing instability), and comorbid health problems. At the conclusion of the interview, we asked participants demographic questions.

We mailed a letter (translated into the non-English languages) signed by each patient's primary care physician inviting the patient to participate in a voluntary 45-minute interview with one of the investigators (KEL), and with an interpreter for the non-English speaking patients, about the patient's experiences obtaining services to prevent colorectal cancer. We offered a $25 cash incentive to participate. Patient interviews lasted one-half to one hour. We conducted the interviews either in the patient's home or in a research office, according to each patient's preference. To interview patients in their primary language, we trained two medical interpreters to assist with the non-English interviews. One interpreter was bilingual in English and in Haitian Creole, and the other interpreter was trilingual in English, Spanish, and Portuguese (including Portuguese Creole).

We also developed an open-ended, semi-structured interview instrument to explore physicians' experiences discussing colorectal cancer screening with their patients. During the interview, we asked each physician the following question: "I recently met with your patient ______. What sorts of things do you think prevented him/her from getting screened?" Prior to each interview, we asked physicians to confirm that they were in fact the patient's primary care physician, and to review the medical record of their patients who had not been screened. The physician interviewer also reviewed each patient's medical record to validate the patient's report of their screening status, and to document any efforts made by the physician to screen the patient. Physicians also completed a brief questionnaire about their demographic characteristics. We offered physicians a $50 cash incentive to participate; interviews lasted one-half to one hour.

### Data analysis

We audiotaped the interviews, with the exception of one patient who declined audiotaping. For that patient, we took detailed notes of the interview. We submitted the audiotapes to an experienced qualitative transcriptionist who transcribed them verbatim. For the non-English language interviews, only the English-language portions (which the interpreter had translated into English) were transcribed. One investigator (KEL) checked the transcripts for accuracy, listening to portions of the audiotape while reading the typed transcription. Two investigators, a primary care physician (KEL) and a medical sociologist (MG) coded and analyzed all of the transcripts. We created codes that reflected patient and physician responses regarding the reasons the patient had not been screened for colorectal cancer. During frequent meetings, we discussed discrepancies in coding and resolved them by consensus. Using the constant comparative method [29], we revised original themes after we compared them with newer themes that emerged in the coding process. We compared themes across cases to ensure that they were both representative and inclusive of all cases. We also compared individual patient and physician perspectives regarding the reasons each patient was not screened. Due to the small sample size, we did not calculate reliability statistics.

## Results

Table 1 shows the demographic characteristics of the 23 patients who were interviewed. Sixteen patients had not received colorectal cancer screening; the remaining 7 patients had either been screened (n = 3) or had received a diagnostic colonoscopy (n = 4) for workup of gastrointestinal symptoms. Most patients were female, non-white, had a low level of education, and had an annual income of less than $15,000. The mean age of participants was 60.9, and nearly all had some form of health insurance. Of the three patients who had undergone colorectal cancer screening, one had a colonoscopy, and 2 were screened by FOBT cards. We also interviewed the 10 primary care physicians of the 16 patients who had not been screened. Half of the physicians were female, most were white (n = 8), and their mean age was 46.

**Table 1:** Patient characteristics (n = 23)

**Characteristics**	
Female (%)	60.9
Mean Age (range)	62.3 (52–74)
Race (%)	
White	34.8
Black	39.1
Other	26.1
Hispanic or Latino origin (%)	26.1
Health Insurance (%)	
Private	4.3
Medicare	21.7
Medicaid	39.1
Free Care^1^	30.4
Uninsured	4.3
Primary Language (%)	
English	34.8
Portuguese/Portuguese Creole	26.1
Spanish	8.7
Haitian Creole	30.4
Education (%)	
Less than high school	47.8
High School only/Vocational/Trade	26.1
College or higher	26.1
Annual Income (%)	
< $5,000	17.4
$5,000–$9,999	34.8
$10,000–$14,999	21.7
≥ $15,000	17.4
Don't know/refused	8.7
Employment (%)	
Employed full or part-time	69.6
Retired	13.0
Unemployed	17.4
Screened for Colorectal Cancer (%)	
Yes	30.4
No	69.6

^1^After being determined ineligible for other payment options, Massachusetts residents can apply for help paying for health center bills from the Massachusetts uncompensated (free) care pool.

Four consistent themes emerged from our analyses of patient interviews: 1) Unscreened patients cited lack of trust in doctors as a major barrier to screening whereas few physicians identified this barrier in their patients; 2) Unscreened patients identified lack of symptoms as the reason they had not been screened; 3) A doctor's recommendation, or lack thereof, significantly influenced patients' decisions to be screened; 4) Patients, but not their physicians, cited fatalistic views about cancer as a barrier. Most patients and physicians cited more than one barrier to screening. Table 2 lists barriers cited most frequently by patients and physicians, respectively. Below we discuss each theme in detail with illustrative verbatim quotations.

**Table 2:** Most frequently cited barriers to colorectal cancer screening identified by patients and by their primary care physicians

Barriers identified by patients	Barriers identified by Physicians
1. Lack of trust (n = 5)	1. Psychosocial issues (n = 6)
2. Lack of symptoms (n = 5)	2. Comorbid medical illness (n = 4)
3. No doctor's recommendation (n = 5)	3. Screening is lower priority (n = 3)
4. Fatalistic views about cancer (n = 3)	4. Patient does not seek preventive care (n = 5)

### Lack of trust

Five of 16 unscreened patients (3 white US-born patients, 1 African-American patient, and one white Portuguese patient from the Azores) cited a lack of trust in doctors as one of the major reasons they had not been screened for colorectal cancer. For example, a white woman from the Azores related that her husband had suffered from oral cancer: "I really don't trust doctors... I really believe that ...all the treatment just helped to kill him faster. So I always tell my daughters if, God forbid...if anything happens to me, I don't want any of that [treatment]." Her physician, a native speaker of Portuguese, recognized that a lack of trust was affecting her patient's decision not to be screened:

"It's a trust issue...She's from the Azores [part of Portugal]... going to the doctor was really a last resort when nothing else, when the local remedies...would not help...Also, we had a dictatorship in Portugal at the time, and there was a secret police as well...people grew up not knowing who to trust...they knew that they were safe within the home, but anything outside of the home was different."

A US-born white man also cited lack of trust in doctors as one of the reasons he had not been screened:

"I have issues around trust, and whether or not things will be done in my best interests...Maybe they want more operations to do. Gee we want to find out if there's more cancer because our surgeons aren't working enough. We can't give enough radiation. We want more business, so we want to look for more cancer."

While this patient's physician did not identify lack of trust as a reason why the patient was unscreened, he did correctly identify psychosocial stressors as another reason the patient had not been screened: "...all I know is he was an [older] guy who was in love with a ... [younger] woman, and was dealing with all those kinds of issues, and had recently left his wife, and it was just sort of a social mess..."

Trust in doctors was a facilitator of screening for 2 of 7 of the screened patients (1 white US-born patient and 1 Spanish-speaking patient from El Salvador). The Salvadoran woman related that she had had a good experience seeing a doctor in El Salvador for a gynecologic complaint. She said of her current primary care doctor: "I'm not planning on stopping seeing her until either I die or something happens to her that she cannot see me again."

### Lack of symptoms

Five of the 16 unscreened patients (2 white US-born patients, 2 African-American patients, and one Portuguese patient from the Azores) cited a lack of symptoms as one of the reasons why they had not been screened, while 4 of the 7 patients who appeared to have been screened had in fact had diagnostic colonoscopies to work up the etiology of gastrointestinal symptoms (2 Portuguese-speaking patients from Brazil, 1 Spanish-speaking patient from El Salvador, and 1 Portuguese-Creole speaking patient from Cape Verde). An unscreened English-speaking white woman told us: "You guys want me to have an examination; I'll tell you something, I have the world's best digestive system..." At the same time, her physician cited comorbid medical illnesses as one reason she had not been screened: "She has really poorly controlled cholesterol and blood pressure, so I imagine it hasn't come up because she's so resistant to taking her blood pressure medicine..." Her physician also said, "I just know her personally, she doesn't want anything done..." A second patient, also an English-speaking white woman, related: "I only go to the doctor if something is hanging off my body or I'm bleeding..." Her physician recalled that she refused to complete FOBT cards, and added:

"I think she was sent to me because she's a psych patient and they sent her down for a primary care physician...I don't recall her having a strong motivation to see a primary care physician and get medical care... I have this vague picture of her as not highly motivated to participate in this medical intervention that was going on and in a hurry to get out and ... I think I felt that I had made a major accomplishment by telling her to go to the dentist."

### A doctor's recommendation

Two of 7 screened patients (both Haitian-Creole speaking patients from Haiti) cited a doctor's recommendation as a facilitator of screening, while 5 out of 16 unscreened patients (1 white US-born patient, 3 Haitian-Creole speaking patients from Haiti, and 1 Spanish-speaking patient from El Salvador) said their doctor had not recommended screening. The importance of a doctor's recommendation, while cited by patients speaking Spanish, English, and Haitian Creole, was especially prominent among Haitian patients. Of the 5 Haitian patients who had not been screened, 3 said they had not been screened because their doctor had not recommended screening. When queried about colorectal cancer screening, one patient replied "I don't know anything about it...the doctor never asked me to do it." Both of the Haitian patients who had been screened reported that they had completed the tests based on their doctor's recommendation.

When we reviewed the medical records of the 5 patients who stated that their doctor had never recommended screening, it appeared that in 2 instances (including one in which the physician was a native speaker of Haitian Creole), a discussion about colorectal cancer screening had indeed taken place, as each physician had referred the patient for a colonoscopy. Most Haitian patients reported that if their doctor recommended screening then they would be screened. One man explained: "Doctor come [sic] after God. After God, it's doctors."

Some physicians were aware that they had not recommended screening, often because of competing priorities – their own priorities or those of the patient. For example, one physician gave the following explanation for why she had not discussed colorectal screening with her patient:

"I think she is a psychologically fragile, a very anxious lady who has been taking care of a disabled husband...And she has been quite overwhelmed. And she's also been dealing with her pulmonary condition which I think has been much more acute. So I think it's a combination of doing a lot of acute stuff and personal issues..."

### Fatalism

Three of 16 unscreened patients expressed fatalistic views about cancer as one of the reasons they had not been screened; two of these patients were African-American men, and the third was a woman of Portuguese descent from the Azores. In each case, the physician was not aware that the patient held fatalistic views about cancer. One African-American man told us: "...Well, I don't want to [get screened for colon cancer] because, hey, you know, I mean, if you got it you got it, but they can't do anything to cure me..." His physician, when asked why the patient was not screened, replied:

"...My impression was that a lot of things got in the way of him getting screened. It seemed like in every progress note, almost every one... the colonoscopy was scheduled, but not done and rescheduled and not done... I know at one point his wife had died. He has a history of substance use, so that interfered. But to what extent his views about colon cancer screening interfered with that, I don't really have a good sense of that..."

Another African-American man, who reported that his father had died from colorectal cancer, related: "I figure if it's [cancer] going to be there it's going to be there. That's the outlook I have." This patient also reported that he was afraid of finding out that he has cancer if he gets the tests done. His physician listed several other reasons why this patient had not been screened. The physician noted a lack of connection between him and the patient, as the patient often consulted other providers at the health center. He also felt that colorectal cancer screening was a lower priority for this patient:

"...There's been the substance abuse issue, there's been a significant musculoskeletal problem... [which] kept him out of work for a long time. Precarious financial, social and home life situation, so all of those things we've been aware of and we've addressed..."

The physician also reported that the patient does not seek preventive care: "He's always come for intermittent complaints or for minor crises in his personal or his medical life. He's never been somebody who seems to have engaged in regular routine health maintenance..." The physician then added, "I don't believe... that anybody actually asked the question about colon cancer."

## Discussion

In this qualitative study of ethnically and linguistically diverse patients receiving care at 8 community health centers in the Boston area, we observed that the 4 following principal factors may prevent patients from being screened for colorectal cancer: distrust of doctors, lack of symptoms, lack of a physician recommendation for screening, and fatalistic beliefs about cancer. Patients of differing race, ethnicity, and language mentioned these factors. It is possible that what these patients have in common – poverty and limited educational attainment – may underlie these barriers to screening.

Why might patients be distrustful of colorectal cancer screening? For African Americans, the legacy of the Tuskegee syphilis experiment and the persistence of health disparities have been shown to decrease their trust in doctors or health care [30]. Others have implicated physicians' interpersonal skills [31] and a lack of continuity in care [32] as contributors to a lack of trust. In our study, several patients trusted their own physician and had regular ongoing care at a community health center. Yet these patients feared that other physicians (such as gastroenterologists, medical oncologists, radiation oncologists and surgeons) would not act in their best interests. It is also possible that fatalistic views about cancer underpin a lack of trust. If a patient believes that a cancer diagnosis will inevitably lead to death, yet their doctor or health care system is promoting an invasive procedure to detect the cancer, the patient may begin to distrust his or her doctor's motives. Why would their doctor promote a test to detect an incurable disease? Perhaps an in-depth discussion with a physician about colorectal cancer screening would help to educate and reassure patients about their concerns. Yet for some patients, physicians cited a preponderance of other medical and psychosocial issues they felt compelled to address, thereby precluding them from conducting even a brief discussion of colorectal screening.

The other barriers to screening we identified, lack of symptoms or of a doctor's recommendation, have been found in a prior study of the U.S. population [9], and our study suggests that these factors may also apply to specific groups that have not been analyzed previously, including Portuguese, Brazilian and Haitian patients. We found that Haitian patients, in particular, cited the lack of a physician recommendation as the main reason they were not screened, and they reported a willingness to comply with any recommendation made by their physicians. Yet when we reviewed these patients' charts, we found that in some cases the physician had in fact recommended colorectal cancer screening. This finding suggests a communication problem during the visit – even when there was no language barrier between a patient and his or her physician. In contrast to prior studies [33], we observed that systems issues, such as long wait times for colonoscopy did not appear to be a major barrier to colorectal cancer screening. It is possible that the barriers we encountered (such as lack of a physician recommendation and lack of trust) lie "upstream" to potential systems issues. If patients are not pursuing screening they are not encountering these systems barriers.

## Conclusion

Our study provides insight into potential barriers to colorectal cancer screening facing disadvantaged patients served by community health centers. A strength of our research is that we included groups in which colorectal cancer screening has not been widely studied, including patients from Haiti, Brazil, the Azores, and Cape Verde. Our study has several limitations. The findings from our sample of poor, ethnically, and linguistically diverse patients who receive care at urban community health centers may not be generalizable to other patient populations. Yet our findings may be generalizable to enclaves of similar patients elsewhere. Our sample was limited to small numbers of patients in each language group, and to a small number of screened patients. Thus, it is unlikely that saturation was reached in our study. We did not back-translate the non-English language translations. However, our physician interviewer spoke fluent Spanish, Portuguese, and French and could assess the validity of the interpreting for most of the non-English language interviews. Our use of qualitative methods precludes us from estimating the prevalence of the barriers we identified among all patients receiving care at community health centers. Finally, interview coding may be subjective.

Disparities in colorectal cancer screening rates according to the race, ethnicity, and socioeconomic status of patients have been well documented [9,10,13,17-19,34,35]. Because community health centers provide care to many disadvantaged patients, these centers are an ideal setting in which to design and implement interventions to improve screening rates. One such intervention might include a community health worker component, where such workers would provide telephone-based outreach to patients identified in the administrative database as not having received screening. Our preliminary findings suggest that it is possible that such interventions could be applied broadly, without substantial tailoring related to the ethnic or linguistic background of patients. Addressing lack of trust in doctors and fatalistic beliefs about cancer – barriers that have not been typically addressed in previous interventions and may occur in all ethnic groups – may improve the success of efforts to promote screening in community health centers. Unburdening primary care physicians of the entire responsibility of addressing all preventive services, perhaps by enlisting the assistance of other members of the health care team, may also increase screening rates.

## Competing interests

The author(s) declare that they have no competing interests.

## Authors' contributions

KEL and MJG conceived the initial study. KEL carried out the interviews and identified themes. All authors helped to refine the analysis, contributed to the final version of the paper and approved the final manuscript.

## Pre-publication history

The pre-publication history for this paper can be accessed here:



## Supplementary Material

Additional File 1 **Semi-structured Interview Schedule: Patient interview.** Click here for file
