# Supplementary material for: Barriers to colorectal cancer screening in community health centers: A qualitative study
Source: BMC Fam Pract. 2008 Feb 27;9:15. doi: 10.1186/1471-2296-9-15 (PMC2373875; doi:10.1186/1471-2296-9-15)
Supplement: Additional File 1 — Semi-structured Interview Schedule: Patient interview. [file 1471-2296-9-15-S1.doc]

## Semi-structured Interview Schedule: Patient interview

*My name is _______, and I am a primary care doctor at one of our neighborhood health centers. I also analyze our hospital data, and have found that a lot of patients aren’t receiving certain tests to prevent cancer. I’m trying to understand why this is the case. I’m really interested in hearing what you have to say about this issue.*

To begin with, I was wondering if you could tell me something about yourself (probes: where you were born, where you grew up, work and family, who live with, involvement in community)?

SES checklist

| Sex | M F |
| --- | --- |
| Age |  |
| Marital Status | Married/Common-law/partner  Widowed  Separated/Divorced  Single/Never Married |
| # of children |  |
| Occupation |  |
| Highest level of Education | Less than high school  High school or equivalent (GED)  Trade/Vocational/Comm. College  College  Beyond college |
| # years of education |  |
| Religion |  |
| Country of birth |  |
| City/town of residence |  |
| Language at home |  |
| Language at work |  |

What is your typical day like? Take today, for instance, what did you do?

**If foreign-born**: What made you come to the United States? What was the health care system like in your country?

How have your experiences with the health care system been here? (probes: insurance status)

Now I’d like to talk to you a bit about cancer prevention. Do you think there are things you can do to prevent cancer? What sorts of things? Do you think it helps to find cancer early? (probe re mammograms and PAPs)

**If seems skeptical of health care system**: Do you think doctors can really do things to help you prevent cancer? In what way? Why do you think that?

Have you or anyone you’re close to ever been told you have cancer? Could you tell me about that?

Have you heard about colon cancer? (If interviewee not familiar with colon cancer, would say: This is the kind of cancer I’m interested in. A lot of people haven’t heard about it-they’ve heard more about other cancers like breast cancer or prostate cancer. It’s basically a cancer that grows in the lower part of your intestine).

Where did you hear about it? (probes: doctor, nurse, family, friends, TV, radio, magazines)

Family history of polyps/colorectal cancer?

Have you ever been checked for colon cancer? How? What was that like for you?

| Ever FOBT |  |
| --- | --- |
| Ever Sigmoidoscopy |  |
| Ever Colonoscopy |  |
| Ever Barium Enema |  |
| Ever Digital rectal exam |  |

What advice would you give to someone who was choosing between the different tests?

**If not screened**:

There are a couple of tests you can do to prevent getting this cancer, or at least find it before it spreads:

Describe FOBT cards-can you see yourself ever doing that?

Describe sigmoidoscopy-can you see yourself ever doing that?

Describe colonoscopy-can you see yourself ever doing that?

What do you think makes some people get these tests done, and others not?

We are trying to figure out how we can encourage patients like you to get checked for colon cancer. How do you think we could do that?

Preferred screening test:

| FOBT |
| --- |
| Sigmoidoscopy |
| Colonoscopy |
| What PCP recommends |
| Unsure |
| Would not be screened |

**Checklists**

Checklist for CRC barriers (if no CRC screening ever):

Feeling like you’re not at risk

No bowel symptoms, such as constipation

Money problems

Having no insurance

Scheduling problems (probe how remembers appointments)

Not able to take time off from work (for colonoscopy)

Worry about complications from the procedure-like a puncture of the intestine

Transportation problems

Not having time

Afraid to find out the results

fears or distrust of the medical system,

pain/discomfort,

feeling violated,

embarrassment,

inconvenience of the preparation,

Symptoms of anxiety (stress, worry, nervousness)

Symptoms of depression (sadness, feeling down)

Substance abuse

Difficulty communicating because don’t speak English

No interpreters

History of trauma

Checklist for facilitators:

PCP mentioned

Family

Friends

Know someone who had colon cancer

Checklist for cultural issues:

go to see doctor/get tests when ill, not for prevention

alternative medicine

shame

being seen as sick or weak

Don’t feel comfortable discussing certain body parts,

Feeling like it’s all out of your control

Checklist for proposed interventions:

letter in the mail, signed by your doctor, stating you were due for colon cancer screening

mailed stool cards

community health worker call you to let you know you were due for colon cancer screening, and to help you to schedule the test.

Community health worker to come with you the day of the test

Health Fair

Community based education (school, church, etc)

Waiting room education

Education after getting mammogram in suite or information about colon cancer in your mammo result letter

Final questions:

| Citizenship |  |
| --- | --- |
| Yearly income, $ | None  <5,000  5,000-9,999  10,000-14,999  15,000-19,999  20,000-29,999  30,000-39,999  40,000-49,999  50,000-59,999  60,000-79,999  > 70,000 |
| Debt |  |
| Which best describes your race (may check off more than 1) | White  Black or African American  American Indian and Alaska Native  Asian  Native Hawaiian and Other Pacific Islander  Other Race: |
| Are you Hispanic or Latino? | Yes No |
| What is your ethnicity (if not Hispanic)? |  |
| Insurance | Medicaid  Free Care  Uninsured  Medicare  Private  Other |

So to summarize, it sounds like the main reason(s) you have/haven’t gotten checked for colon cancer is/are ________________________________. Is that correct?

Is there anything else that you think is important for me to know about you?
